# Highly efficient phosphor-glass composites by pressureless sintering

**DOI:** 10.1038/s41467-020-16649-z

**Published:** 2020-06-04

**Authors:** Dao Zhang, Wenge Xiao, Chang Liu, Xiaofeng Liu, Jinjun Ren, Beibei Xu, Jianrong Qiu

**Affiliations:** 1 State Key Laboratory of Modern Optical Instrumentation, School of Optical Science and Engineering, Zhejiang University, Hangzhou, Zhejiang 310027 China; 2 School of Materials Science and Engineering, Zhejiang University, Hangzhou, Zhejiang 310027 China; 3 Key Laboratory of Materials for High Power Laser, Shanghai Institute of Optics and Fine Mechanics, Chinese Academy of Sciences, Shanghai, 201800 China; 4 CAS Center for Excellence in Ultra-intense Laser Science, Shanghai Institute of Optics and Fine Mechanics, Chinese Academy of Sciences, Shanghai, 201800 China

**Keywords:** Design, synthesis and processing, Inorganic LEDs

## Abstract

The development of high-power white light-emitting diodes demands highly efficient and stable all-inorganic color converters. In this respect, phosphor-glass/ceramic composites show great promise as they could combine the merits of high quantum efficiency of phosphors and high chemical and thermal stabilities of glass/ceramic matrices. However, strong interfacial reaction between phosphors and matrices at high temperature results in quantum efficiency loss of the embedded phosphors, and traditional solutions rely on high-pressure consolidation techniques. Here we report the intrinsic inhibition of interfacial reaction by using silica glass rather than multicomponent glasses as the matrix. The embedment of phosphors is achieved via a pressureless sintering method, rendering these color-tunable phosphor-glass composites not only accessible to three-dimensional printing technique, but also highly efficient (internal quantum efficiency >90.0%), thermally stable at 1200 °C and hydrothermally stable at 200 °C. Our results provide a facile and general strategy for developing all-inorganic functional composites.

## Abstract (web summary)

Phosphor-glass/ceramic composites are attractive for high-power white light-emitting diodes, but interfacial reaction leads to loss of quantum efficiency. Here the authors report a reduction sintering method for embedment of phosphors into silica glass with limited interfacial reaction.

## Introduction

Color converters that can emit light of desired wavelengths after absorbing incident light play a key role in light-emitting^1–3^ and -detecting^4^ devices as well as photovoltaics^5,6^. However, conventional color converters with luminescent particles embedded in transparent organic polymers are notoriously easy to degrade during exposure to heat, moisture and short-wavelength radiation, leading to much shorter lifetime of the devices than expected^7,8^. Recently, the so-called phosphor-converted white light-emitting diodes (pc-WLEDs) have been widely used in general lighting and display backlighting, but their extension to high-power and high-brightness lighting fields such as street lighting, automotive headlamp and large-size display, is extremely sluggish^1–3,9,10^. Besides thermal quenching of phosphors^1,3,9,11^, unacceptable degradation of organic resins under intense blue/near-UV radiation and high temperature (>150 °C) becomes a major obstacle^7–10^.

Traditional doped single-phase (or eutectic) transparent ceramics^12,13^ and bulk crystals^14^ are efficient all-inorganic color converters whereby organic encapsulation could be circumvented, but the costly preparation and the very limited variety exclude them from mass production and general application^9^. In contrast, directly consolidating the mixtures of commercial phosphors and glass/ceramic powders into bulk composites is believed to be a much more feasible and economical strategy^10^^,^^15–19^, partly because of the diverse selection of phosphors and matrix compositions. Generally, more thermally stable matrix requires higher sintering temperature for full densification, whereas strong interfacial reaction with matrix at high temperature (>800 °C) will corrode the phosphor particles. As in other composite materials^20–22^, the most fundamental issue here is how to preserve the intactness of the embedded phosphors so that the composites could retain the luminescence properties of the raw phosphors. Many researchers have sought to inhibit the interfacial reaction by adopting low-melting glasses (such as low-silica^16,19,23^, tellurite^17,18^ and phosphate^24,25^ glasses) and ceramics (CaF_2_^26^ and hydroxyapatite^27^) as the matrix, and/or resorting to high-pressure and high-vacuum techniques like hot isostatic pressing^25^, spark plasma sintering (SPS)^27,28^ and gas pressure sintering^29^. However, high quantum efficiency (QE) of phosphors is seldom retained by these strategies even though they have sacrificed the high chemical and thermal stabilities and the facile synthesis^10^. Moreover, those reported matrices suffer from certain disadvantages including low visible transparency^17,24–27^, high price^17,18^ and heavy metal (for example, Pb and Sb) containment^30,31^. On the other hand, high-pressure consolidation techniques not only require sophisticated equipment, but also are limited to manufacturing plate-like objects, so that three-dimensional (3D) structure, for example the preferred hemispherical dome^32–34^, is hardly realized for all-inorganic color converters. Hence, the development of color converters that are highly efficient, chemically and thermally stable, cost-effective, and easy to shape remains an important but challenging task.

Silicon (Si) is the second most abundant element in the earth’s crust, and is nontoxic. Fused silica (SiO_2_) glass, known for optical fibers, possesses outstanding chemical and thermal stabilities as well as high visible transparency, possibly being an excellent matrix for phosphors. Nevertheless, the ultra-high melting temperature (>1700 °C) makes efficient phosphors in silica glass (PiSG) composites only accessible to expensive SPS systems by far^28^. Here we report the facile synthesis of PiSG by a reduction sintering method, where amorphous silica nanoparticles are utilized to facilitate the densification process and develop photocurable composite slurries. Through exploiting the temperature- and composition-dependent solid solubility of Si^4+^ in phosphors, we find that interfacial reaction between phosphors and silica glass could be well suppressed even at the temperature of 1250 °C. We further demonstrate that the perfect combination of phosphors and silica glass with negligible interfacial reaction produces a series of highly efficient (for Y_3_Al_5_O_12_:Ce (YAG:Ce) in silica glass, internal QE (IQE) = 95.0%), chemically and thermally stable phosphor-glass composites. By virtue of pressureless sintering, we also realize the freeform fabrication of all-inorganic color converters for the first time by combining with modern 3D printing technology.

## Results

### Design and synthesis of YAG:Ce-PiSG

Despite extensive research in developing new phosphors for pc-WLEDs^1–3,11^, the yellow phosphor YAG:Ce is still the dominant one owing to its suitable excitation and emission spectra, high IQE, and superior chemical and thermal stabilities^35^. Therefore, as a demonstration, we first designed and synthesized YAG:Ce-PiSG. The crystal structure of YAG:Ce is thermally stable up to 1700 °C^36^, and its IQE can be well maintained after heat treatment for 3 h at 800 °C in air (Supplementary Fig. 1), but YAG:Ce-glass/ceramic composites sintered at 700 °C for less than 30 min show obvious IQE loss^19,26,27^, suggesting that using low-melting materials is not an effective way to inhibit interfacial reaction. Among various glass systems, silicate glasses with high silica content are the optimal matrix materials for their high chemical and thermal stabilities. Large amounts of glass modifiers like Na^+^, Zn^2+^ and Ca^2+^ are usually added to reduce the melting point^19,23,30^, which, in turn, will cause intrinsically more severe corrosive reaction of glass matrix with phosphor owing to the high reactivity and diffusivity of those low-valence ions^10,37^ and the presence of non-bridging oxygen atoms^18^. Particularly, while the flexibility in composition of garnet structure allows high co-solubility of low-valence ions and Si^4+^ in YAG^34^, the solubility of Si^4+^ in YAG is very low (<0.1%) due to the size and charge mismatches between Si^4+^ and Al^3+^/Y^3+^^38^. Hence, we reasoned that interfacial reaction could be essentially inhibited by embedding YAG:Ce into pure silica glass instead of multicomponent glasses.

**Fig. 1:**
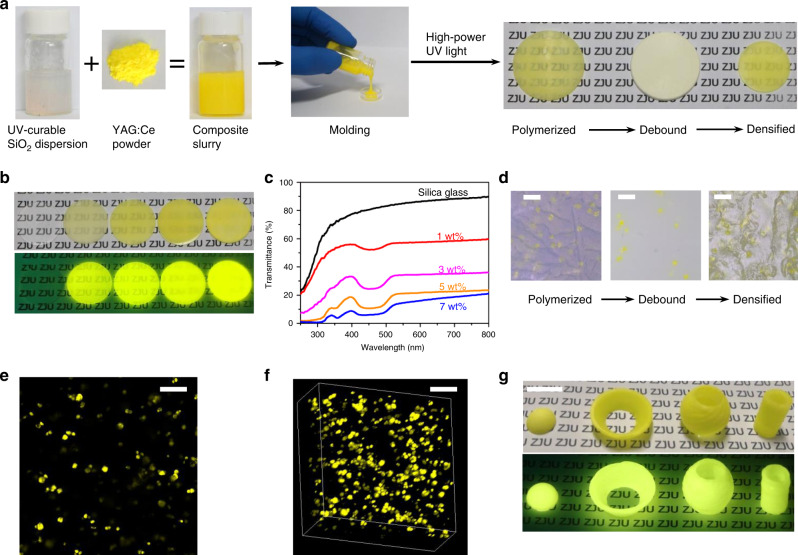
Fabrication of YAG:Ce-PiSG. **a** Amorphous silica nanoparticles are first dispersed into UV-curable monomer, and then YAG:Ce powders are mixed with the UV-curable dispersion to form the slurry, which is put into the mould and polymerized into flat discs under high-power 365 nm light. After debound and sintered, they are fully densified into bulk composite (1.8 cm in diameter). Note that the resultant slurry shows no obvious phosphor settlement after siting for 30 min. **b** Flat discs of YAG:Ce-PiSG with various YAG:Ce contents (from left to right: 0, 1, 3, 5 and 7 wt%, 1.8 cm in diameter and 1.0 mm in thickness). **c** Total transmittance of YAG:Ce-PiSG with various YAG:Ce contents (1.0 mm in thickness). **d** Fluorescence microscope images of YAG:Ce/silica composite after polymerized, debound and densified (scale bar, 100 μm). **e** Surface CLSM image of 3 wt% YAG:Ce-PiSG (scale bar, 100 μm). **f** 3D reconstruction CLSM image of 3 wt% YAG:Ce-PiSG (scale bar, 100 μm). **g** Examples of printed and sintered 3 wt% YAG:Ce-PiSG (scale bar, 1 cm).

Previous investigation on YAG transparent ceramics has shown that the sintering aid SiO_2_ starts to react with YAG in the vicinity of 1400 °C^39^. To lower the densifying temperature away from 1400 °C and simultaneously avoid the use of high pressure and high vacuum, we chose amorphous silica nanoparticles with high sintering activity as the precursor to facilitate the densification process. A large quantity of amorphous silica nanoparticles (53.7 wt%) are fully dispersed into a UV curable monomer mixture based on 2-Hydroxyethyl methacrylate (HEMA) to form a low-viscosity silica dispersions^40^, and then YAG:Ce powders are added to obtain the final composite slurry where YAG:Ce particles can be well suspended (Fig. 1a). The slurry is then polymerized into transparent flat-plate green bodies within 30 s under high-power UV light. Green bodies with complex 3D structure can be also rapidly produced from the developed photocurable slurry with stereolithography (SLA) 3D printing (Supplementary Fig. 2), by which Kotz et al have recently fabricated various silica glass structure parts^41,42^. After two-step heat treatment for debinding and densifying (Methods), the green bodies become porous and then are fully densified into transparent pure silica glass or translucent yellow YAG:Ce-PiSG (Fig. 1a,b), where for PiSG doped with 1 wt% YAG:Ce (1 wt% YAG:Ce-PiSG) the linear shrinkage is 4.7 and 27.9% after debound and densified, respectively.

As shown in Fig. 1c, pure silica glass exhibits excellent transparency with a total transmittance of 80.5% at the wavelength of 450 nm (86.2% at 600 nm), implying that full densification is achieved. With incorporating YAG:Ce into silica glass, the as-synthesized samples become translucent, accompanied by intense yellow emission upon 450 nm excitation (Fig. 1b). Transparency reduction is mainly attributed to the enhanced light scattering due to the different refractive indices of YAG:Ce (1.84) and silica glass (1.46); nevertheless, moderate light scattering is beneficial to the color uniformity and the light extraction of high-power pc-WLED, especially when laser diodes (LDs) are used as the excitation source^9^. Fluorescence microscope images (Fig. 1d) indicate that the size of YAG:Ce particles remains constant (10–20 μm) as YAG:Ce/silica composite is polymerized, debound and densified. A confocal laser scanning microscope (CLSM) with a 488 nm laser is employed to investigate the distribution of YAG particles inside silica glass. It can be clearly seen from the surface and 3D reconstruction CLSM images (Fig. 1e,f) that bright yellow points are uniformly dispersed in silica glass. The quick UV curing used here greatly alleviate the phosphor sedimentation, an intractable problem existing in the heat-curing process of the sol-gel method (>12 h)^43^ as well as conventional organic encapsulation (1.5 h)^44^.

To accomplish rapid manufacturing of YAG:Ce-PiSG with complex shapes and inspired by the sintering of transparent ceramics in pure hydrogen atmosphere^45^, we adopted a reduction (5%H_2_ + 95%N_2_) sintering method that are usually used for preparing phosphor powders, to quicken the densifying process and protect Ce^3+^ of YAG:Ce-silica composite from oxidation at high temperature. In addition to its convenience and cost-effectiveness, another significant advantage of this strategy over those pressure-assisted methods is maintaining the original shapes of green bodies after densification. Accordingly, by combining with the SLA 3D printing technique^41^, we realized the 3D construction of YAG:Ce-PiSG (see Fig. 1g), which is extremely difficult for traditional tableting-sintering and melting-quenching methods^10^. It takes only 74 min for our desktop 3D printer to print one batch (18 pieces) of dome-type parts (Supplementary Fig. 2) and the printing time could be further reduced via optimizing the printing conditions. With the development of 3D printing techniques, rapid manufacturing of color converters with complex shapes will trigger off the modular assembly and mass customization of WLED lamps; for example, size-compatible dome-types YAG:Ce-PiSG can directly replace the polycarbonate lens of blue LED chip (1 W) to form a WLED device (Supplementary Fig. 3).

### Performance characterizations of YAG:Ce-PiSG

**Fig. 2:**
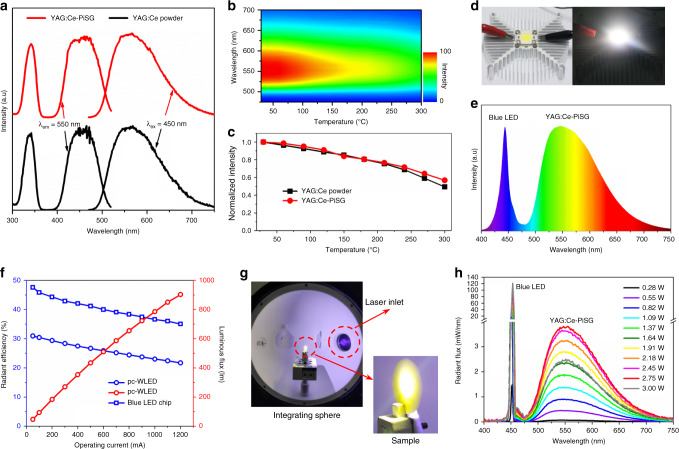
Optical characterization of YAG:Ce-PiSG flat discs. **a** Excitation and emission spectra of 5 wt% YAG-PiSG and YAG:Ce powder. **b** Temperature-dependent emission spectra of 5 wt% YAG:Ce-PiSG. **c** Temperature dependences of the integrated emission intensity of 5 wt% YAG:Ce-PiSG and YAG:Ce powder. **d** Photos of the as-fabricated pc-WLED (10 W) (left) and the lighted one in the darkness (right). **e** Electroluminescence spectrum of pc-WLED under the current of 50 mA. The correlated color temperature (CCT), the CIE coordinates and the color rendering index (*R*_a_) is 4586 K, (0.3702, 0.4378) and 62, respectively. **f** The operating current dependences of the luminous flux of pc-WLED and the radiant efficiencies of blue LED and pc-WLED. **g** The experimental setup in the integrating sphere for characterizing the tolerance of high-density blue radiation of 5 wt% YAG:Ce-PiSG. Note that no heat sink is used here. **h** Luminescence spectra of 5 wt% YAG:Ce-PiSG (1 cm in diameter, 0.6 mm in thickness) under various laser optical powers.

As shown in Fig. 2a, the excitation and emission spectra of YAG:Ce-PiSG are the same with the corresponding YAG:Ce powder. Notably, benefiting from the high transparency of silica glass in the UV range, the excitation band around 341 nm of YAG:Ce-PiSG is as high as that of YAG:Ce powder, which is absent or largely suppressed in those based on low-melting glasses/ceramics^17,24–27^. Under 450 nm excitation, the IQE of 5 wt% YAG:Ce-PiSG is determined to be as high as 95.0% (for YAG:Ce powder, IQE = 97.6%, Supplementary Table 3), meaning that nearly no IQE loss occurs when YAG:Ce is embedded into silica glass. To the best of our knowledge, the as-synthesized YAG:Ce-PiSG is one of the most efficient all-inorganic color converters including transparent ceramics and single crystals. As with YAG:Ce powder, YAG:Ce-PiSG shows fluorescence thermal quenching (Fig. 2b) at elevated temperature, and the decline of the integrated emission intensity of YAG:Ce-PiSG is consistent with that of YAG:Ce powders, retaining higher than 80% of the initial value at 150 °C (Fig. 2c). The unimpaired luminescence properties of YAG:Ce-PiSG imply that the intactness of YAG:Ce is well preserved when YAG:Ce-silica composite are consolidated into bulk materials at high temperature (1250 °C).

High-temperature sintering naturally grants YAG:Ce-PiSG a superior thermal stability. To confirm this unique feature, we baked YAG:Ce-PiSG at the temperature of 1200 °C in air for 10 h. The IQE and the appearance of YAG:Ce-PiSG are both unchanged, while those low-melting inorganic materials phosphors not only suffer from obvious IQE loss but also soften or even melt at much lower temperature (Supplementary Fig. 4). For fully estimating the moisture resistance at elevated temperature, a highly accelerated temperature and humidity test is necessary. Here we adopted a modified immersion test for YAG:Ce-PiSG where the sample was immersed in distilled water and then hydrothermally treated for 10 h, which is much harsher than that for traditional optical glasses^46^. Although those low-melting inorganic materials (except phosphate glasses) are thermally stable at <300 °C, all of them are moisture sensitive, especially when the temperature is higher than 100 °C (Supplementary Fig. 4), which is insufficient to achieve ultra-long lifetime (ideally, 100,000 h) of pc-WLED devices. In comparison, YAG:Ce-PiSG is hydrothermally stable at 200 °C, manifesting its satisfactory chemical and thermal stabilities for high-power LED applications. Another essential parameter of matrix materials for color conveters is the thermal conductivity because if the huge amount of generated heat during LED operation cannot be dissipated quickly, it will give rise to strong thermal quenching of phosphors. YAG:Ce-PiSG has the thermal conductivity of 1.44 and 1.93 W m^−1^ K^−1^ at room temperature and 250 °C, respectively, which is eight times that of organic resins (∼0.2 W m^−1^ K^−1^) and also higher than that of those multicomponent glasses (for example, 0.71 W m^−1^ K^−1^ for tellurite and phosphate glasses)^17,24^. These results indicate that YAG:Ce-PiSG combines the excellent luminescence properties of YAG:Ce with the outstanding chemical and thermal properties of silica glass, without sacrificing the easiness of fabrication.

To demonstrate the performance of YAG:Ce-PiSG in practical LED applications, we fabricated a high-power (10 W) pc-WLED device (Fig. 2d), and the electroluminescent spectrum is shown in Fig. 2e. The luminous efficiency (LE) is as high as 119 lm W^−1^ at 50 mA, and the luminous flux can reach 785 lm at 1000 mA with LE decreasing to 86 lm W^−1^ due to the so-called “efficiency droop” of the LED chip under high operating current (Fig. 2f)^47^. This performance can be further improved by optimizing the scattering effect inside the PiSG composite and using blue LED chip with higher radiant efficiency. In order to evaluate the capability to withstand high-density blue radiation, we focused a power-tunable 455 nm laser on the YAG:Ce-PiSG plate without any heat sink (Fig. 2g). The luminescence saturation for YAG:Ce-PiSG occurs at the optical power density of 2.72 W (3.46 W mm^−2^) (Fig. 2h), which is six times higher than that (0.5 W mm^−2^) for those multicomponent silicate glass matrices^9,19^. In contrast to silicone and those low-melting materials that cannot survive at higher than 1 W mm^−2^^19^, owing to the high chemical and thermal stabilities, YAG:Ce-PiSG could serve as a good color converter for laser-driven lighting if heat sink like sapphire plate^9^, is added.

### Structural evidences for inhibited interfacial reaction

**Fig. 3:**
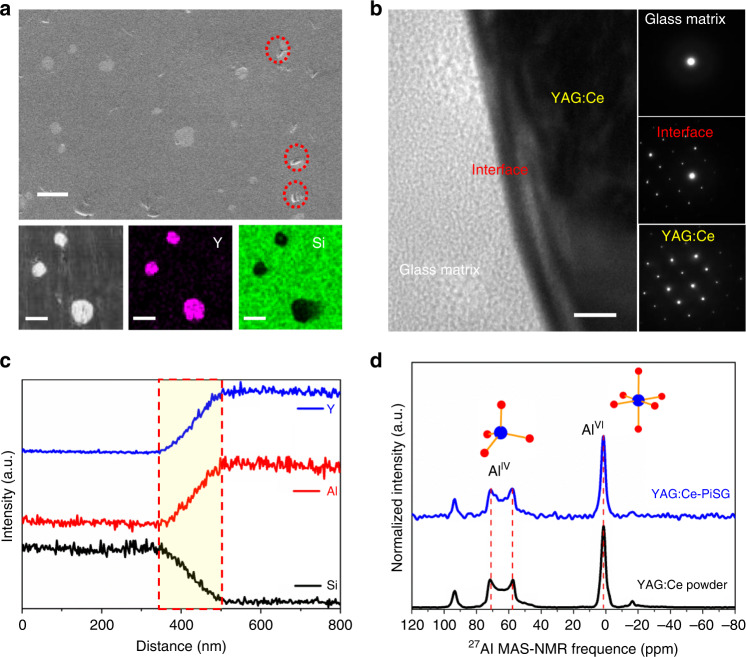
Structural evidences for limited interfacial reaction between YAG:Ce and silica glass. **a** SEM (top) and EDS mapping (down) images of YAG:Ce-PiSG (scale bar, 25 μm). Red circles denote the possible existence of pores. **b** TEM bright-field image (left) and SAED patterns (right) of YAG:Ce-PiSG (scale bar, 50 nm). **c** TEM-EDS line scanning of YAG:Ce-PiSG. **d** Solid-state single-pulse ^27^Al MAS-NMR spectra of YAG:Ce powder and 5 wt% YAG:Ce-PiSG. Asterisk (*) represents spinning sidebands and pound sign (#) satellite transitions.

Although the incorporation of YAG:Ce induces the precipitation of silica glass into cristobalite phase, multicomponent crystalline phase signifying the strong admixture of SiO_2_ with YAG:Ce cannot be detected by the powder X-ray diffraction (XRD) patterns (Supplementary Fig. 5). We first used the scanning electron microscopy (SEM) coupled with energy-dispersive X-ray spectroscopy (EDS) to analyze the microstructrure of YAG:Ce-PiSG. As demonstrated by the SEM images and EDS analysis (Fig. 3a and Supplementary Table 2), YAG:Ce-PiSG has a smooth surface with a very limited number of pores, and Y clusters with a diameter of 10–20 μm are distributed in the Si matrix, confirming the successful preparation of YAG:Ce-PiSG. To study the interface reaction between YAG:Ce particles and glass matrix at nanometer scale, we conducted transmission electron microscopy (TEM), selected area electron diffraction (SAED) analysis and TEM-EDS line scanning measurements for YAG:Ce-PiSG. The TEM image in Fig. 3b shows a clear boundary representing the interface between YAG:Ce and glass matrix, which is evidenced by the concurrence of the diffraction spots belonging to amorphous SiO_2_ and YAG:Ce crystal in the SAED pattern of the interface. In the TEM-EDS element spectra (Fig. 3c), the abrupt but opposite changes in the content of Y/Al and Si elements denote the interficial reaction zone, and the thickness of the interface of YAG:Ce-PiSG is thus estimated to be about 150 nm. In comparison, this thickness is about 200 nm for YAG:Ce-PiG based on low-melting silicate glasses despite a much shorter holding time (30 min)^48,49^.

Solid-state nuclear magnetic resonance (NMR) spectroscopy is a powerful tool to provide atomic structural information in material science. Figure 3d shows the ^27^Al magic angle spinning NMR (MAS-NMR) spectra of YAG:Ce powder and YAG:Ce-PiSG. The single-peak signal at 1.5 ppm and the well-defined quadrupolar peak at 71.3 ppm can be clearly resolved, where the former represents six-coordinated Al (Al^VI^) and the latter four-coordinated Al (Al^IV^). The maximum peak positions, i.e. the average isotropic chemical shifts (δ_iso_), as well as the peak shapes of the two aluminum species show no difference in both spectra, indicating that the local environment of these polyhedrons remains nearly the same after YAG:Ce are embedded into silica glass. The ratios of the integrated signal intensities of Al^IV^ and Al^VI^ species from YAG:Ce powder and YAG:Ce-PiSG are estimated to be 42.3:57.8 and 41.5:58.5, respectively, both of which agree well with the expected 2:3 ratio for YAG crystal. In addition, the ^29^Si MAS-NMR spectra of pure silica glass and YAG-PiSG (Supplementary Fig. 6) are almost identical, which is consistent with the results from ^27^Al MAS-NMR spectra. Moreover, the long-term (10 h) high-temperature stability of YAG:Ce-PiSG at 1200 °C also reflect the intrinsically inhibited interfacial reaction between YAG:Ce and silica glass. Our results unambiguously confirm that interfacial reaction between YAG:Ce and silica glass matrix is very limited even at 1250 °C for 3 h.

### Versatile phosphor-silica glass composites

The extremely small diffusion rate of highly charged Si^4+^ and the absence of non-bridging oxygens^18,28^ largely improve the activation energy (namely the required temperature) for the interfacial reaction of phosphor with silica glass, as compared with multicomponent glasses. Moreover, a reduction atmosphere is employed not only to assist the densification process, but also to preserve Ce^3+^ and Eu^2+^, two most widely used activators in phosphors, which otherwise would be oxidized into luminescence killers (Ce^4+^ and Eu^3+^) at high temperature. Using amorphous silica nanoparticles lower the required temperature to only 1250 °C, but, nevertheless, the structure of most phosphors including oxides and oxynitrides are thermally stable at this temperature. Therefore, the proposed strategy can generally be applied to achieve color tunable emission in silica glass based composites.

**Fig. 4:**
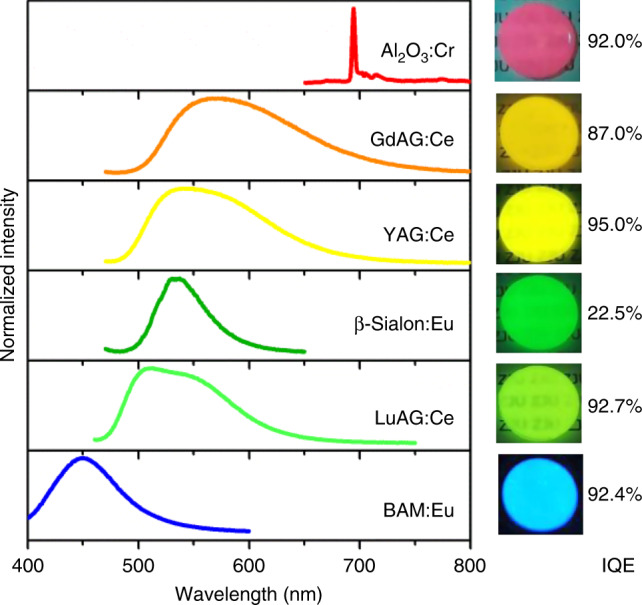
Emission spectra of PiSG embedded with various phosphors. The emission color of these PiSG composites can cover the whole visible range when doped with different phosphors. Photos of PiSG samples under the 365 nm (for BAM:Eu) or 450 nm (for others) excitation and their IQE values are both shown on the right.

As shown in Fig. 4, not only garnet-type phosphors including Lu_3_Al_5_O_12_:Ce (LuAG:Ce, blue-green), (Gd,Y)_3_Al_5_O_12_:Ce (GdAG:Ce, orange) as well as YAG:Ce (yellow), but other well-known phosphors such as BaMgAl_10_O_17_:Eu (BAM:Eu, blue), β-SiAlON:Eu (β-Sialon:Eu, green) and Al_2_O_3_:Cr (far-red) were particularly incorporated into silica glass for exemplification (Fig. 4). As expected, all the as-synthesized phosphor-glass composites are highly efficient with limited IQE loss except the β-Sialon:Eu doped one (Supplementary Table 3). Unlike α-sialon, β-sialon is structurally unstable at high temperature due to N_2_ evaporation^43^, resulting in large IQE loss. Using smaller amorphous silica nanoparticles (<15 nm) or tetramethoxysilane as the precursor^43,50^ could reduce the required temperature to lower than 1100 °C. It is thus anticipated that more kinds of phosphors could be incorporated into silica glass without obvious IQE loss. However, the densifying temperature of silica glasses is still too high for red nitride phosphors, and this limitation cannot be well circumvented until novel oxide or oxynitride red phosphors with high thermal stability are developed. Finally, silica glass acts as an effective buffer for the embedded phosphors against oxidation at high temperature (Supplementary Fig. 4), which will endow them with better performance as the thermographic phosphor for high-temperature (>1000 °C) thermometry^36^.

## Discussion

In summary, we have developed a reduction sintering method for rapid producing highly efficient phosphor-silica glass composites. Such a pressureless consolidation technique is compatible with modern 3D printing technique, thus enabling the 3D construction of all-inorganic color converters that are thermally stable up to 1200 °C. We confirmed that interfacial reaction between phosphors and silica glass is intrinsically inhibited without adding any glass modifier cations, and the resultant composites possessing excellent luminescence properties, shows great potential for high-power and high-brightness pc-WLEDs. Our work provides a facile and general synthetic strategy to color-tunable bulk composites based on silica glass for various light-emitting and light-detecting applications, which also have strong implications for developing innovative glass based composite materials with high-temperature stability^51^.

## Methods

### Materials

HEMA (99%), tetra(ethylene glycol) diacrylate (TTGDA), Diethylene glycol dibenzoate (DEDB, 99.5%), 2,2-dimethoxy-2-phenylacetophenone (DMPA, 99%) and Sudan red G (95%) were purchased from Aladdin, China. Tinuvin 1130 (Basf, Germany). Amorphous silica nanoparticles (a mean diameter of 40 nm, Aerosil OX50) were purchased from Evonik, Germany. Except Al_2_O_3_:Cr^3+^ prepared by ourselves, all other phosphors are commercially available from phosphor manufacturers (β-Sialon:Eu only from Mitsubishi Chemical, Japan).

### Moulding and 3D printing of green bodies

Figure 1a shows the detailed manufacturing process of YAG:Ce-PiSG. 28.1 wt% HEMA, 14.5 wt% DEDB and 3.7 wt% TTGDA were thoroughly mixed. Then, 53.7 wt% amorphous silica nanoparticles were added to the monomer mixture in 100 times and stirred with an agitator (D500 DragonLab) after each addition to form the dispersion. Afterwards, 0.4 wt% DMPA was added into the dispersion (with respect to the amount of reactive monomer). Note that for SLA 3D printing 0.02 wt% Sudan red G was added. Finally, YAG:Ce powder (1–7 wt% with respect to the amount of silica nanoparticles) was mixed into the dispersion, which was defoamed using a vacuum ball mill for 1 h. After that, a homogeneous slurry containing silica nanoparticles and YAG:Ce powders was obtained. For disc-like plates of green bodies, the photopolymerization was performed under a high-power UV lamp (365 nm, 1000 W, 273 mW cm^−2^) for 25 s.

In the 3D printing process, a desktop LCD SLA 3D printer (L1121, isun3d, China) was used. It has a 3.5-inch LCD screen with a resolution of 2560 × 1140 DPI and an array of 405 nm LEDs as the light source. 3D models for printing were designed using SolidWorks (Dassault Systèmes, France) software and exported in the STL file format. The STL files were then imported into the 3D printing software (ChiTu Slicer, China) for slicing and printing. The printing process is automatic and the printing time of one batch depends on the height of the designed parts and the scanning speed of UV light. Finally, the printed green bodies were immersed into isopropyl alcohol for two minutes to remove the surface attachment. To demonstrate the modularization of pc-WLEDs, the size of the hemispherical dome of YAG:Ce-PiSG was designed according to mid-power (1 W) 450 nm LED chip so that the polycarbonate lens can be directly replaced by the as-prepared YAG:Ce-PiSG and sealed with heat conducting silicone (Supplementary Figs. 2 and 3).

### Sintering of YAG:Ce-PiSG

The green bodies were first heat-treated at 600 °C in air for 6 h with a heating rate of 1 °C min^−1^ using a box furnace (KSL-1100X, HF-Kejing, China). Then, they were sintered at 1250 °C in reducing atmosphere (5%H_2_ + 95%N_2_) (0.1 L min^−1^) for 3 h with a heating rate of 3 °C min^−1^ in a high-temperature tube furnace (GSL-1400X, HF-Kejing, China). Finally, the obtained YAG:Ce-PiSG was polished for measurements. Other phosphors embedded PiSGs were synthesized by the same procedures with YAG:Ce-PiSG.

### Characterization

The transmittance spectra were measured with a UV-vis-infrared spectrometer (U-4100, Hitachi, Japan). Fluorescence microscope image was recorded on the fluorescence microscope (BX53, Olympus, Japan) in the bright-field mode. The distribution of phosphor particles within silica glass was analyzed by using a confocal laser scanning microscope (TCS SP5, Leica, Germany). The emission, excitation and temperature-dependent emission spectra were measured on FLS920P spectrometer (Edinburgh Instruments, UK), where the temperature was controlled by a high-temperature fluorescence test device (TAP-02, Orient KOJI). The IQE and Absorption were measured with a UV-NIR absolute photoluminescence quantum yield spectrometer (Quantaurus-QY Plus C13534-12, Hamamatsu Photonics, Japan), where the error is within 1%. The thermal conductivity measurement was performed by a physical performance measurement system (PPMS, Quantum Design DynaCool-9). All the high-temperature stability tests were conducted in air with a tube furnace. The chemical stability was characterized by a highly accelerated temperature and humidity stress test that the samples are immersed in distilled water and hydrothermally treated at higher than 100 °C for 10 h using a stainless-steel reactor with a Teflon lining, which is much harsher than the standard D85 test (85 °C and 85% relative humidity) as well as the normal hydrolytic resistance test for optical glass^46^. The XRD patterns were obtained on a powder XRD spectrometer (D/MAX 2550/PC, Rigaku, Japan). The SEM images were taken with a scanning electron microscope (Utral-55, Carl Zeiss, Germany), and a layer of gold was sputtered to the surface of the sample to enhance the conductivity. The elementary composition (EDS and EDS mapping) was analyzed by an energy-dispersive spectrometer (INCA Energy Coater, Oxford Instruments) coupled to the SEM. TEM image, SAED pattern and EDS line scanning were acquired on a JEM-2100 (JEOL, Japan) operating at 200 kV accelerating voltage. The sample was thinned using the TESCAN GAIA3 that integrates a field emission SEM with a focused ion beam (FIB). Solid-state NMR spectra were obtained on a Bruker Avance III HD 500 M spectrometer (11.7 T). ^27^Al MAS-NMR spectra were recorded at a resonance frequency of 130.3 MHz (^27^Al) using a 4 mm MAS-NMR probe at a spinning rate of 12 kHz. The pulse length was 0.56 μs (10°). The relaxation delay was 64 s for YAG:Ce-PiSG, and 40 s for YAG:Ce powder. The chemical shifts of ^27^Al were referenced to an aqueous solution of 1 M Al(NO_3_)_3_. A resonance frequency of 99.3 MHz, a spinning rate of 8 kHz and a pulse length of 2 μs (30°) were adopted to acquire the ^29^Si MAS NMR spectra. The relaxation delays of silica glass and YAG:Ce-PiSG were set as 350 s, and the chemical shifts were referenced to tetrakis(trimethylsilyl)silane.

### LED/LD Device fabrication and measurement

High-power pc-WLED devices (Fig. 2d) were fabricated by combining 10 W 450 nm LED chips (Bridgelux, USA) with 5 wt% YAG:Ce-PiSG (0.8 mm in thickness). A power adjustable 455 nm LD (LSR455CP-FC-12W, Lasever, China) was used as the excitation source to evaluate the capability of YAG:Ce-PiSG to withstand high-density radiation. As shown in Fig. 2g, the laser was focused directly on 5 wt% YAG:Ce YAG:Ce-PiSG plate (1 cm in diameter, 0.6 mm in thickness) to form a prototype white LD device, where the beam is nearly circular and the spot size was fixed at a diameter of 1.0 mm (0.785 mm^2^). The optical output powers of the blue LD under different input currents were measured with a laser power meter (PM100D, Thorlabs). The optical properties of white LED/LD devices including the electroluminescence spectra, *R*a, CCT, and LE were measured by an integrated test system (LHS-1000, EVERFINE) including photoelectric characteristic testing system, high accuracy array spectrophotometer (HAAS-2000), stabilized DC power supply, and an integrating sphere (SPEKTRON R98, Φ 80 cm).

## Supplementary information

Supplementary Information

Peer Review File

## Data Availability

All the data supporting the findings in this study are available in the paper and Supplementary Information. Additional data related to this paper are available from the corresponding authors upon request.
