# Supplementary material for: Highly efficient phosphor-glass composites by pressureless sintering
Source: Nat Commun. 2020 Jun 4;11:2805. doi: 10.1038/s41467-020-16649-z (PMC7272639; doi:10.1038/s41467-020-16649-z)
Supplement: Supplementary file 1 — Supplementary Information [file 41467_2020_16649_MOESM1_ESM.pdf]

Supplementary Information for

Highly efficient phosphor-glass composites by pressureless sintering

Zhang et al.

Supplementary Figures

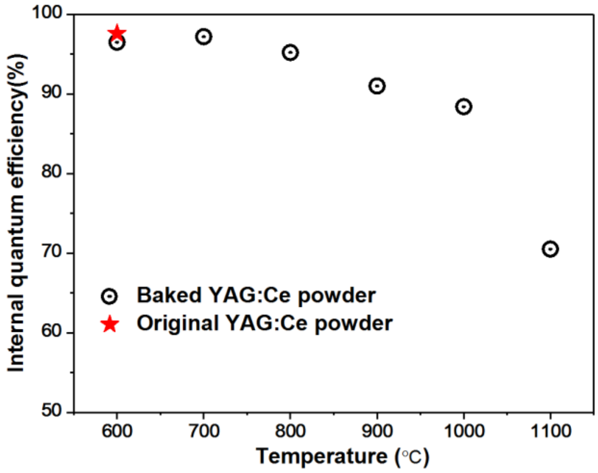

Supplementary Fig. 1 Internal quantum efficiency of YAG:Ce powders after baked at different temperatures

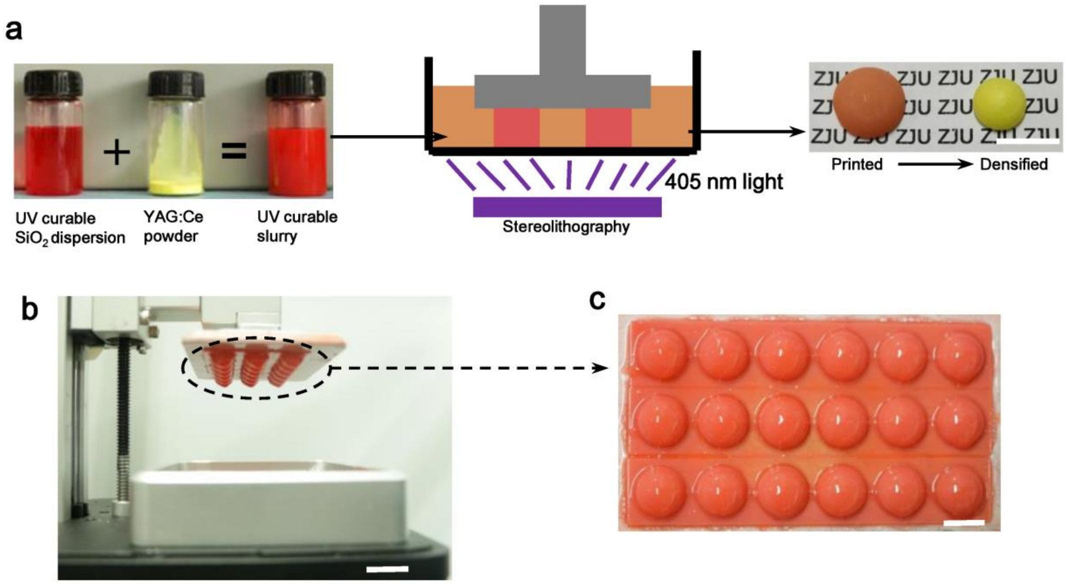

Supplementary Fig. 2 3D printing of YAG:Ce-PiSG. (**a**) YAG:Ce powders are first mixed with the UV-curable dispersion consisting of UV-curable monomer (Sudan red G is added as the light absorber) and amorphous silica nanoparticles, and then the as-formed slurry is used for printing dome selected as the exemplary shape in a SLA system. The resulting green bodies are densified into YAG:Ce-PiSG after debound and sintered (scale bar, 1 cm). (**b**) One patch of dome-type parts on platform of 3D printer (scale bar, 2 cm). (**c**) One patch of freshly printed dome-type parts (scale bar, 1 cm).

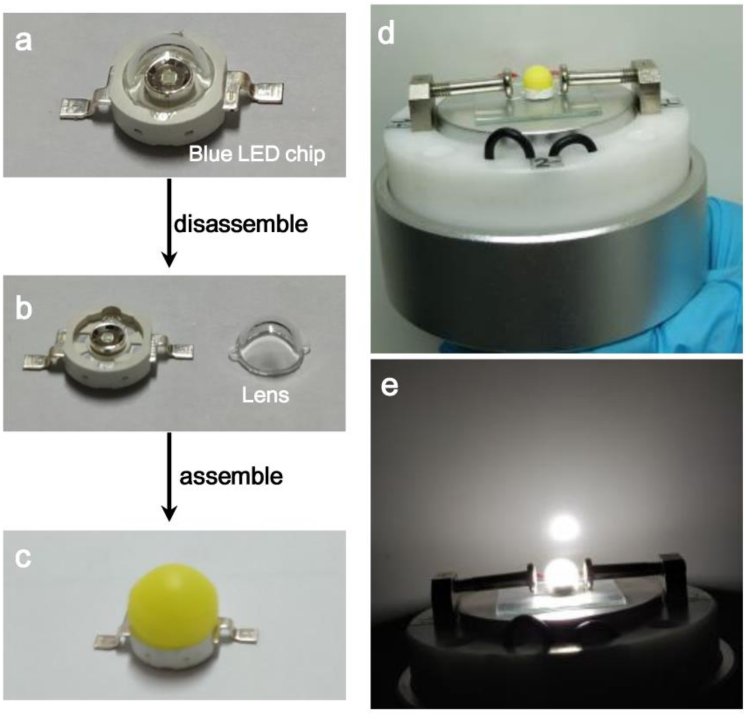

Supplementary Fig. 3 Demonstration of modular assembly of pc-WLEDs. A typical mid-powder blue LED (**a**) is disassembled into a LED chip and a dome-like polycarbonate lens (**b**). Then, the LED chip is directly capped with dome-like YAG:Ce-PiSG sealed by the heat-conducting adhesive (**c** and **d**). Lighted WLED device in the darkness is also shown (**e**).

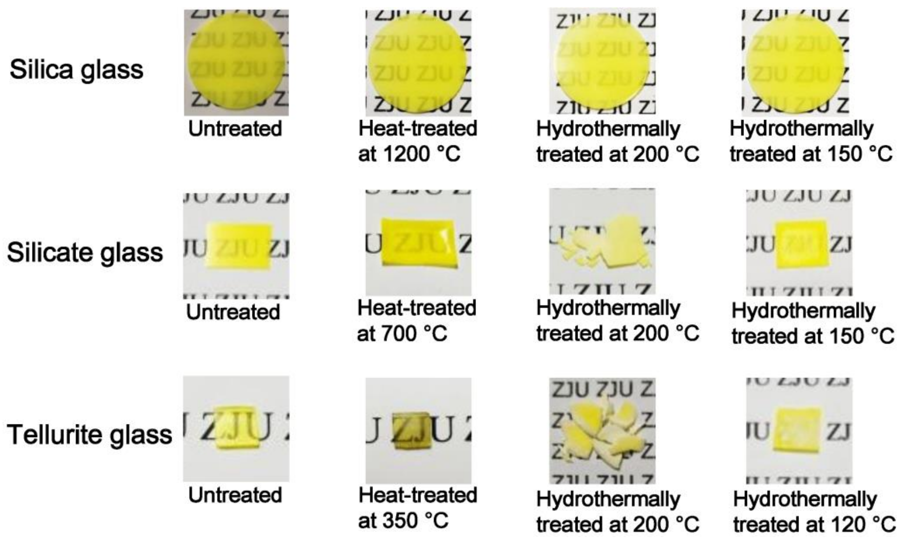

Supplementary Fig. 4 Comparison of the chemical and thermal stabilities of YAG:Ce-PiG based on different glass matrices. Heat-treatment in air for 10 h: the appearance and IQE of YAG:Ce-PiSG show no change after heat-treated at 1200 °C; YAG:Ce-PiG based on soda-lime silicate glass deforms obviously at 700 °C and loses 15% of IQE at 500 °C; YAG:Ce-PiG based on tellurite glass becomes black at 350 °C and lose 80% of IQE, and will melt at 400 °C. Hydrothermal treatment for 10 h: the appearance of YAG:Ce-PiSG shows no change at 200 °C, while YAG:Ce-PiG based on soda-lime silicate glass starts to dissolve at 150 °C, and for tellurite glass, 120 °C. Note that PiG based on tellurite glass (TeO_2-Sb_2O_3-B_2O_3-ZnO-BaO-Na_2O) was synthesized by ourselves according to Supplementary Ref.[1], and PiG based on silicate gass (SiO_2-Na_2O-CaO) was brought from Bright Phosphor Composites Technology, China.

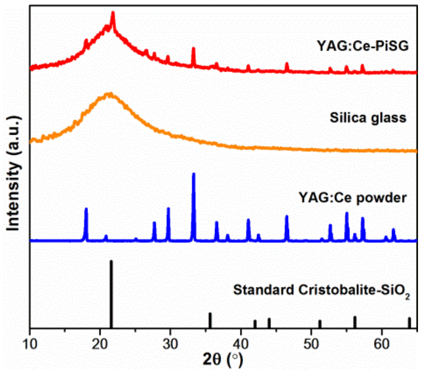

Supplementary Fig. 5 XRD patterns of YAG:Ce powder, silica glass and 5 wt% YAG:Ce-PiSG.

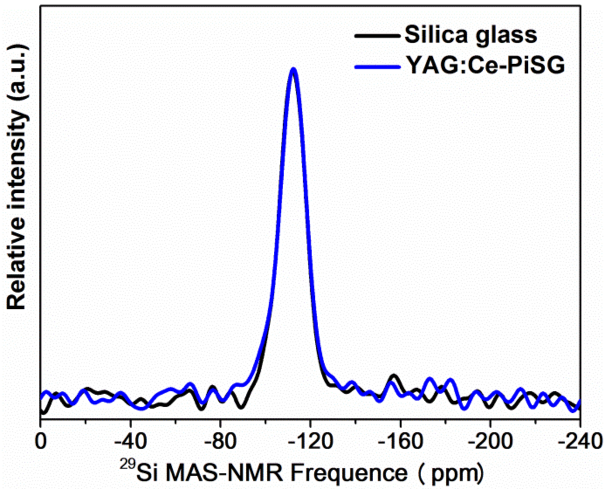

Supplementary Fig. 6 ^{29}Si MAS-NMR spectra of silica glass and 5 wt% YAG:Ce-PiSG. The two ^{29}Si spectra are almost identical, further confirming that there is very limited Al entering SiO_2 phase, consistent with the results from ^{27}Al spectra.

Supplementary Tables

Supplementary Table 1 IQE and absorption of YAG:Ce-PiSG and YAG:Ce powder.

YAG:Ce content in PiSG (wt%)	IQE (%)	Absorption (%)
1	91.8	73.3
3	92.4	82.4
5	95.0	84.1
7	93.6	86.3
YAG:Ce powder	97.6	64.3

Supplementary Table 2 EDS analysis of YAG:Ce-PiSG.

Element	wt%	at%
YAG:Ce region		
C	48.86	66.97
O	23.35	24.02
Al	9.19	5.61
Y	18.00	3.33
Ce	0.60	0.07
Total	100	100
Matrix region		
C	40.15	50.22
O	43.96	41.28
Si	15.89	8.50
Total	100	100

The sample was glued by conductive carbon tape.

Supplementary Table 3 IQE and absorption of phosphors and the as-synthesized phosphor-silica glass composites.

Samples	Excitation wavelength (nm)	IQE (%)	Absorption (%)
YAG:Ce powder	450	97.6	63.7
5 wt% YAG:Ce-PiSG	450	95.0	84.1
LuAG powder	450	92.4	67.1
5 wt% LuAG:Ce-PiSG	450	92.7	77.9
GdAG powder	450	90.8	67.1
5 wt% GdAG:Ce-PiSG	450	87.0	68.0
β-Sialon:Eu	450	79.4	55.9
3 wt% β-Sialon:Eu-PiSG	450	22.5	62.0
BAM:Eu powder	380	96.0	51.3
3 wt% BAM:Eu-PiSG	380	92.4	34.6
Al_2O_3:Cr powder	420	97.1	45.4
3 wt% Al_2O_3:Cr-PiSG	420	92.0	52.7

Supplementary References

1. Lin, H. et al. Phosphor-in-glass for high-powered remote-type white AC-LED. ACS Appl. Mater. Inter. **6,** 21264-21269 (2014)
