# Supplementary material for: Highly efficient phosphor-glass composites by pressureless sintering
Source: Nat Commun. 2020 Jun 4;11:2805. doi: 10.1038/s41467-020-16649-z (PMC7272639; doi:10.1038/s41467-020-16649-z)
Supplement: Supplementary file 2 — Peer Review File [file 41467_2020_16649_MOESM2_ESM.docx]

Peer Review Information

**Journal:** Nature Communications

**Manuscript title:** Highly efficient phosphor-glass composites by pressureless sintering

**Corresponding author name(s):** Dr Wenge Xiao

**Editorial notes:**

**none**

**Reviewer comments & decisions:**

**Reviewer comments, first version:**

Reviewers' comments:

Reviewer #1 (Remarks to the Author):

In this manuscript, the authors report the synthesis of fully dense phosphor glass-ceramics using pressureless sintering. They start from a mixture of a crystalline phosphor (YAG for example) and silica glass. Interestingly the authors note the absence of interfacial reaction between the aluminate crystalline phase and the pure silica matrix. The resulting material exhibits high internal quantum efficiency and good thermal stability. Moreover, the process enables to access original shapes and possible 3D printing.

Overall the manuscript is well written and presented. It is an original and sound piece of work which deserves publication. Please find below a few comments and suggestions which may help to improve the quality of the manuscript:

- The authors claim that their PiSG samples are translucent. They also state that moderate light scattering is beneficial to color uniformity and light extraction. This is true but the transparency of their samples strongly drops as soon as YAG is included in the glass matrix. This should be state more clearly. Moreover the thickness of the samples should be given, especially in the figure captions. If the authors wish to increase the transparency, it might be interesting to consider the addition of GeO2 in the glass matrix. I guess that the overall behavior may remain the same (even though the price would increase and thermal stability would decrease) but as GeO2 exhibits a much larger refractive index than SiO2, the refractive index difference between YAG and the glass might enable better transparency in the final sample.
- The authors did not comment the effect of the size of the YAG crystals used for sample preparation. This would be an interesting point to develop and correlate with the final transparency. Also commenting the effect of HEMA, DEBB and TTGDA content variations in the synthesis process would help further reproduction of the process.
- The authors state that the internal quantum efficiency is very high. This is fine but they do not comment much on the overall quantum yield. Given the low amount of YAG crystals in the glass-ceramic samples (especially required to ensure light output) this yield remains limited compared to pure YAG ceramics or YAG-Al2O3 ceramics with which the authors compare their materials in the introduction section. What is the optimum thickness and YAG content in order not to decrease too much the translucency? I guess that the thickness cannot be too large to avoid opacity (no light going out). This could be discussed in more details. Also the authors state that "the as-synthesized YAG:Ce-PiSG is the most efficient among the reported all-inorganic color converters including transparent ceramics and single crystals". Could they propose a reason for that?
- Regarding the thermal conductivity, once again the authors compare their materials with organic resins and multicomponent glasses. To be fair they should also compare it with pure YAG transparent ceramics or single crystals. Same remark for the luminescence saturation measurements.
- The use of solid state NMR spectroscopy is interesting to investigate both the YAG crystals and silica glass. However this study could be more developped. Currently the authors show that 27Al MAS-NMR spectra match the expected YAG phase. This is not surprising but it would be interesting to improve the statistics of measurements and see if any secondary signal can be detected, for exmaple correpsonding to some limited Al which is now in the glass. At the moment the spectrum looks a bit noisy to do so. Also 29Si NMR experiments would be complementary to see if some Si entered in the YAG structure (see S.Alarache et al., JPCC, 115, 20499, 2011). Also advanced 27Al and 29Si NMR coupling experiments probing spatial/bonding interactions could be interesting.
- Can very large pieces of YAG PiSG be synthesized? Or is there any problem with cracks during the sintering process. Overall the problem of cracks appearance is not discussed in the paper whereas it would be interesting to know how these appears depending on the various conditions that the authors have tried.
- In figure 3 the authors present EDS mapping (SEM) of Y and Si. Using such a map only provides a general view of the sample. This is fine to illustrate the overall idea but more details could be provided to see (1) small deviation form the ideal view (Si in YAG or Y or Al in SiO2 - currently not seen given the chosen scale - a line composition variation would probably provide more details) and (2) what happens at higher magnification (can interfacial reaction be detected between SiO2 and YAG at the nanometer/atomic scale (TEM)?
- The fact that SiAlON is not as stable as the other examples might also be related to the fact that there is Si in the crystalline phase.

Minor comments:
- The acronym (PiSG) should be developed at the first occurrence of the Phosphor in silica glass expression.
- In the "Performance characterizations..." section: replace "As shown in Fig. 4a" by "As shown in Fig. 2a"

In conclusion the manuscript is a nice piece of work of which I recommend publication. Hopefully the manuscript can be further improved by answering the above comments/suggestions.


Reviewer #2 (Remarks to the Author):

The manuscript suggests a new method to fabricate a phosphor in glass using nano-sized silica powders to embed phosphors. It seems to be reasonably written but the followings should be addressed or discussed before consideration of publication.

1. They suggested that their method is cost-effective process compared to other previous methods. It could be true for phosphor in silica glass (PiSG) fabricated by SPS process. However, their method still require quite high sintering temperature of 1250 oC along with reducing atmosphere. When compared with commercial phosphor in glass (PiG) using silicate glass which is sintered at 750 oC without atmospheric control, the present method require much higher temperature and needs special facility to control atmosphere as well, demanding higher production cost. Even considering the cost of gas pressured sintering (GPS) for the densification of commercial PiG, it is hard to believe that the present method could be cost effective in mass production when considering the cost of nano-sized silica glass powders.

2. Although the authors suggested that the present PiSG showed high transparency, the highest transparency was ~ 80% in visible range for the sintered SG without phosphor. As they mentioned, it is due to the residual pores within the sintered silica glass. Since the pores cannot be avoided without vacuuming or external gas pressure during the sintering process, it is hard to maintain the same color coordinate in mass production, which is why commercial PiG goes under additional GPS process. They need to discuss how they can control the amount of pores and their distribution within the silica glass matrix for reproducible mass production. Moreover, in order to suggest the advantage of the present pressure-less method, they need to compare color conversion properties with a PiG which used silicate glass and GPS process.

3. As previous studies also reported PiSG with other methods such as sol-gel or SPS sintering, it seems reasonable to compare their data with the present method to clearly demonstrate their novelty.

4. Although they successfully fabricated PiSG with various phosphors, due to high sintering temperature of 1250 oC, only limited phosphors which have very high thermal stability can be applied. For example, as they indicated, beta-SiAlON showed degradation of IQE. This suggests that their method cannot incorporate other phosphors such as CaAlSiN-based red phosphors and thus their application could be limited.

5. The authors suggested that the reaction between SiO2 and YAG has been suppressed based on SEM/EDS and NMR results. However, other previous results which used silicate glasses also showed no apparent reaction layers between the glass matrix and phosphors based on SEM/EDS results. Moreover, since reaction is mostly restricted in the interface region and NMR signal gives averaged effect within the composite, it is hard to identify the possible reaction between the glass and phosphor by monitoring Al-NMR. It seems the phosphor-glass reaction has been suppressed since it was sintered under reducing atmosphere, but in order to clearly claim it, it seems to show TEM result as reported previously (Shyu et. al, J. Am. Ceram. Soc., 100, 1460-1471 (2017)).

**Author rebuttal, first version:**

**Response to Reviewer #1:**

Dear Reviewer:

Thank you very much for your very constructive and valuable comments on our manuscript. According to your suggestions, we have made relevant revision. The revised sentences were highlighted with red font color in the revised manuscript.

**General comment:** In this manuscript, the authors report the synthesis of fully dense phosphor glass-ceramics using pressureless sintering. They start from a mixture of a crystalline phosphor (YAG for example) and silica glass. Interestingly the authors note the absence of interfacial reaction between the aluminate crystalline phase and the pure silica matrix. The resulting material exhibits high internal quantum efficiency and good thermal stability. Moreover, the process enables to access original shapes and possible 3D printing. Overall the manuscript is well written and presented. It is an original and sound piece of work which deserves publication. Please find below a few comments and suggestions which may help to improve the quality of the manuscript.

**Question 1:** The authors claim that their PiSG samples are translucent. They also state that moderate light scattering is beneficial to color uniformity and light extraction. This is true but the transparency of their samples strongly drops as soon as YAG is included in the glass matrix. This should be state more clearly. Moreover the thickness of the samples should be given, especially in the figure captions. If the authors wish to increase the transparency, it might be interesting to consider the addition of GeO_2_ in the glass matrix. I guess that the overall behavior may remain the same (even though the price would increase and thermal stability would decrease) but as GeO_2_ exhibits a much larger refractive index than SiO_2_, the refractive index difference between YAG and the glass might enable better transparency in the final sample.

**Reply 1:** **First**, as we have discussed in our manuscript, the transparency reduction of PiSG compared with pure silica glass is mainly due to the difference in refractive index between silica glass (1.46) and YAG:Ce (1.86), which also exists in the traditional phosphor-in-silicone composites; generally, stronger backscattering of the emitted light will result in lower luminous efficiency in the phosphor-converted white LEDs (pc-WLEDs), and in this respect the more transparent, the better; nevertheless, recent studies have shown that luminous efficiency can be also improved by optimizing the morphology (spherical geometry is preferred) and volume fraction (1~5%) of the scatterers (pores or second phase), and the thickness of the color converters (***Ref.:*** *Opt. Express 2015, 23, A1629-A1641; Opt. Lett. 2019, 44, 479-482*); moreover, for high-power pc-WLEDs using high-power blue LED or LD as the excitation source, if the light scattering is not sufficiently high, the incident blue light and the converted yellow light cannot be mixed well and the light extraction efficiency is also low because of the high total-internal reflection (***Ref.:*** *Proc. SPIE, 2014, 9190, 91900C*); besides, the scattering is indispensable to reduce the adverse speckle in the laser-driving lighting (***Ref.:*** *Appl. Opt. 2017, 56, 278-283*); as a result, the commercialized phosphor-glass composites based on low-melting silicate glasses have small transparency (<30%, 0.2~0.4 mm in thickness) such as Lumiphous^TM^ ([*https://www.neg.co.jp/en/rd/topics/product-lumiphous/*](https://www.neg.co.jp/en/rd/topics/product-lumiphous/)) (Nippon Electric Glass, Japan) and phosphor in glass composites (Bright Phosphor Composites Technology, China) ([*http://www.bpc-t.cn/PR2/13.html*](http://www.bpc-t.cn/PR2/13.html)), and to the best of our knowledge, translucent ceramic converter is also adopted in practical application (***Ref.:*** *US patent:* *US20180011393*); we believe the optimization of the scattering effect is a systematic engineering. **Second**, all the flat discs of our PiSGs are about **1.0 mm in thickness** (and 1.8 cm in diameter), which have been given in the caption of Fig. 1 according to your suggestion. **Third**, we completely agree with you that the addition of GeO_2_ could increase the transparency of PiSG, which, however, will largely increase the cost and deteriorate the thermal stability as you have pointed out. In addition, amorphous GeO_2_ nanoparticles have not been yet commercially available, which will further increase the cost (Note that the price of amorphous SiO_2_ nanoparticles is lower than 30 dollar/kg). Actually, several researchers have tried to add TiO_2_, TeO_2_ and SnO_2_ to increase the refractive index in the sol-gel method, but the coloration of these glass matrices is hard to avoid (***Ref.:*** *US patent: US20130306911A1;* *J. Ceram. Soc. Jpn. 2013, 121, 361–365*). Anyway, how to tune the refractive index of glass matrix without sacrificing other properties is another meaningful research topic.

**Question 2:** The authors did not comment the effect of the size of the YAG crystals used for sample preparation. This would be an interesting point to develop and correlate with the final transparency. Also commenting the effect of HEMA, DEBB and TTGDA content variations in the synthesis process would help further reproduction of the process.

**Reply 2: It is true that** the size of YAG:Ce particles can affect the transparency of PiSG only if it is comparable to the wavelength of incident light (< 1 μm) according to light scattering theory. However, the typical median size of the commercialized phosphors is around 10~20 μm as smaller size (especially < 1 μm) will result in lower quantum efficiency. Therefore, considering the possibility for practical application, we used the most commercial available YAG:Ce particles which are both cheap and with high quantum efficiency. In the Methods part of our manuscript, we have given the optimal contents of HEMA, DEBB and TTGDA, and our results can be easily reproduced by others if they follow the procedures given in our paper. Besides, we would like to recommend the readers to refer to some previous papers if they want to further optimize or even modify the synthesis (***Ref.:*** *J. Eur. Ceram. Soc. 2011, 31, 2221–2229*; *Adv. Mater. 2016, 28, 4646–4650*) as well as ***Ref. 40 and 41*** in our manuscript.

**Question 3:** The authors state that the internal quantum efficiency is very high. This is fine but they do not comment much on the overall quantum yield. Given the low amount of YAG crystals in the glass-ceramic samples (especially required to ensure light output) this yield remains limited compared to pure YAG ceramics or YAG-Al2O3 ceramics with which the authors compare their materials in the introduction section. What is the optimum thickness and YAG content in order not to decrease too much the translucency? I guess that the thickness cannot be too large to avoid opacity (no light going out). This could be discussed in more details. Also the authors state that "the as-synthesized YAG:Ce-PiSG is the most efficient among the reported all-inorganic color converters including transparent ceramics and single crystals". Could they propose a reason for that?

**Reply 3: W**e think you are concerned with external quantum efficiency (EQE) of our PiSGs since EQE represents the overall conversion ability taking into account both of the absorption (Abs) and internal QE (IQE). In **Supplementary Table 1**, we have given the absorption as well as the IQE of PiSGs with different contents of YAG:Ce; for example, for **1wt%YAG:Ce-PiSG**, Abs=73.3%, IQE=91.8%, then **EQE =67.3%**; for **5wt%YAG:Ce-PiSG**, Abs=84.1%, IQE=95.0%, then **EQE =79.9%**; for **YAG:Ce powder**, Abs=64.3%, IQE=97.6%, then **EQE =62.8%**; please note that the Abs of YAG:Ce powder is relatively low because of the smaller refractive index of air (1) than silica glass (1.46), and the Abs will be enhanced when YAG:Ce powder is embedded into silicone. As regards transparent/translucent YAG:Ce ceramics, nearly all of them were fabricated by high-vacuum reactive sintering (1700 °C or higher), so that plenty of defects such as oxygen vacancies and residual stress are inevitable and they should be post-annealed in air (around 1400 °C); consequently, some of Ce^3+^ ions will be finally oxidized into Ce^4+^ ions, famous luminescence killer for the emission of Ce^3+^; therefore, those transparent/translucent YAG:Ce ceramics usually have only about 0.1 mol% of Ce^3+^ to avoid the Ce^3+^/Ce^4+^ pairs, thus achieving high IQE (90~95%) (***Ref.****: J. Mater. Chem. C 2019, 7, 4057-4065; J. Mater. Chem. C 2019, 7, 11449-11456*). As with transparent/translucent YAG:Ce ceramics, YAG:Ce single crystals have small Ce^3+^ concentration (~0.1 mol%) because of the strong segregation of Ce^3+^ in YAG single crystal; besides, a significant part of the generated light is trapped within YAG crystal and finally lost owing to high total-internal reflection (***Ref.****: Jpn. J. Appl. Phys. 2002, 41, L887–L888; Opt. Express 2016, 24, 251040; Mater. Res. Express 2015, 2, 055503*). Note that it is very difficult to introduce suitable light scattering inside the single crystal, although the light extraction efficiency as well as angular color uniformity can be enhanced by producing various micro/nano structures on the surface of single crystals (***Ref.****: Opt. Express 2016, 24, 17522*). In addition, Ce^3+^ segregation has been also evidenced in transparent YAG:Ce ceramics (***Ref.:*** *Opt. Mater. 2011, 33, 684–687*). Considering that YAG:Ce powder typically has 2 mol% Ce^3+^, the overall quantum yield (you are concerned with) of our PiSGs are comparable to these YAG:Ce transparent ceramics or single crystals. To be more strictly stated, we also have changed “the most efficient among the reported all-inorganic color converters including transparent ceramics and single crystals” to “one of the most efficient all-inorganic color converters including transparent ceramics and single crystals”, since YAG:Ce transparent ceramics or single crystals could have high IQE at low Ce^3+^ doping concentration. Finally, the optimum thickness of PiSG for practical LED/LD applications not only depends on the content of phosphors in PiSG, but also the performance requirements of light sources, because the luminous efficiency (LE) is strongly related to the spectrum distribution of the white light, *i.e.* correlated color temperature (CCT) and color rendering index (CRI or *R*_a_), as shown in **Table R1**. All in all, before entering the market there is much optimization work to be done.

**Table R1.** Electroluminescence properties of the as-fabricated LED devices under the current of 100 mA.

| YAG:Ce content (wt%) | Thickness (mm) | LE (lm/W) | CCT (K) | *R*_a_ |
| --- | --- | --- | --- | --- |
| 1 wt% | 0.8 mm | 74 | 10,0000 | 77 |
| 3 wt% | 0.8 mm | 106 | 4938 | 64 |
| 5 wt% | 0.8 mm | 118 | 4631 | 62 |
| 7 wt% | 0.8 mm | 107 | 4122 | 60 |
| 5 wt% | 0.4 mm | 97 | 6287 | 68 |
| 5 wt% | 0.6 mm | 107 | 4994 | 64 |
| 5 wt% | 1.0 mm | 90 | 3871 | 59 |

**Question 4:** Regarding the thermal conductivity, once again the authors compare their materials with organic resins and multicomponent glasses. To be fair they should also compare it with pure YAG transparent ceramics or single crystals. Same remark for the luminescence saturation measurements.

**Reply 4:** It is well known that the major advantage of pure YAG transparent ceramics or single crystals is the high thermal conductivity (5~10 W/m/K) (***Ref.****: Nat. Commun. 2018, 9, 1175*) and glasses inherently possess relatively low thermal conductivity (~1.46 W/m/K for silica glass). Accordingly, the luminescence saturation is about 10~14 W/mm^2^ for YAG:Ce (0.2~0.4 mol% Ce^3+^) transparent ceramics (***Ref.****:* *J. Mater. Chem. C, 2019, 7, 11449; Laser Photonics Rev. 2019, 13, 1900147*) and 3.46 W/mm^2^ for our 5wt%YAG:Ce-PiSG. However, the price of YAG transparent ceramics or single crystals is at least two orders of magnitude higher than that of their powder counterparts (embedded in organic resins), which is acceptable only in several high-end products such as high-grade cars and the cinemas. As we have discussed in the introduction part of our manuscript, PiG composites could serve as an alternative strategy for extending high-power WLEDs to general applications because of their facile synthesis and much lower cost. Moreover, unlike low-melting multicomponent glasses, pure silica glass used here possesses excellent chemical and thermal stabilities, which provides the possibility of applying to laser-driven lighting if appropriate heat sink like sapphire plate (for transmissive configuration) and aluminum plate (for reflective configuration) is added (***Ref.:*** *J. Mater. Chem. C, 2019, 7, 354; Adv. Optical Mater. 2017, 5, 1700347*). Besides, PiSG composites outperform YAG transparent ceramics or single crystals in the reproducibility and mass customization, which is highly desired for high-power WLED lamps.

**Question 5:** The use of solid state NMR spectroscopy is interesting to investigate both the YAG crystals and silica glass. However this study could be more developed. Currently the authors show that 27Al MAS-NMR spectra match the expected YAG phase. This is not surprising but it would be interesting to improve the statistics of measurements and see if any secondary signal can be detected, for example corresponding to some limited Al which is now in the glass. At the moment the spectrum looks a bit noisy to do so. Also 29Si NMR experiments would be complementary to see if some Si entered in the YAG structure (see S. Alarache et al., JPCC, 115, 20499, 2011). Also advanced 27Al and 29Si NMR coupling experiments probing spatial/bonding interactions could be interesting.

**Reply 5: First,** the content of YAG:Ce is only 1~7 wt% in our YAG:Ce-PiSG samples, and our XRD patterns as well as luminescence measurements have indicated that interfacial reaction between YAG:Ce particles and silica glass is very limited; in addition, the longitude relaxation time of ^27^Al is very long (20 s); therefore, the signal of ^27^Al looks a bit noisy for YAG:Ce-PiSG; the ^27^Al spectrum of silica glass is almost identical with that of YAG:Ce powders, implying that there is no detectable secondary signal. **Second,** we have also measured the ^29^Si spectra of pure silica glass and YAG-PiSG (**Fig. R1**) according to your suggestion; the two ^29^Si spectra are almost identical, further confirming that there is very limited Al entering SiO_2_ phase, consistent with the results from ^27^Al spectra. **Third,** in the paper (***Ref.:*** *J. Phys. Chem. C 2011, 115, 20499–20506*), the content of YAG is as high as 96 mol% in the glass-ceramic, and SiO_2_ (4 mol%) is isotopically ^29^Si-enriched (98%) silica, so that the ^27^Al{^29^Si} D-HMQC is possible and necessary to probe the spatial interactions; however, the Si element in amorphous silica nanoparticles used here is natural abundance; even in the long time ^27^Al single pulse spectra we cannot find the detectable ^27^Al signal from the glasses, not to mention the ^27^Al{^29^Si} D-HMQC experiment; the efficiency of ^27^Al{^29^Si} D-HMQC is much less than that of ^27^Al single pulse experiment. **Finally,** as suggested by you in **Question 7**, we have performed high-resolution transmission electron microscope (HRTEM) measurements to give more clear evidence of the limited interfacial reaction, and please see **Reply 7** for details**.** (**Fig. R1** is also added in the revised manuscript)

**Fig. R1.** **^29^Si MAS-NMR spectra of silica glass and 5 wt% YAG:Ce-PiSG.**

**Question 6:** Can very large pieces of YAG PiSG be synthesized? Or is there any problem with cracks during the sintering process. Overall the problem of cracks appearance is not discussed in the paper whereas it would be interesting to know how these appears depending on the various conditions that the authors have tried.

**Reply 6:** It is theoretically possible to synthesize large-sized YAG:Ce-PiSG by our method since it is free from both of high vacuum and high pressure. Nevertheless, we should be aware that like other sintered inorganic bulk materials, the quality of large-sized PiSG samples strongly depends on the temperature uniformity inside the furnace chamber given the green bodies are well prepared. Using a laboratory high-temperature tube furnace (the size of tube: 60 mm in diameter and 1000 mm in length), we can easily obtain 20~30 mm in diameter and 1~2 mm in thickness, which are large enough for most of LED/LD applications. No cracks can be found in nearly all the as-synthesized PiSG plates, and if the green bodies are not well heat-treated, sometimes we can find several cracks in the sintered 3D printed samples (see **Fig. R2**). The latter is mainly attributed to the nonuniform stress distribution of curved green bodies and the limited printing resolution (a few tens of micrometers) of our desktop LCD SLA 3D printer. The whole printing and sintering process deserves to be further optimized before realizing the modular assembly and mass customization of WLED lamps.

**Fig. R2.** **Enlarged photos of PiSGs doped with 3wt% YAG:Ce.** (**a**) Flat disc sample with a thickness 1.0 mm (scale bar, 2 mm); (**b**) Flat disc sample upon 455 nm excitation (scale bar, 2 mm); (**c**) Printed dome-type sample without any polishing (scale bar, 1 mm). several cracks perpendicular to the direction of layer-by-layer printing process can be observed.

**Question 7:** In figure 3 the authors present EDS mapping (SEM) of Y and Si. Using such a map only provides a general view of the sample. This is fine to illustrate the overall idea but more details could be provided to see (1) small deviation from the ideal view (Si in YAG or Y or Al in SiO2 - currently not seen given the chosen scale - a line composition variation would probably provide more details) and (2) what happens at higher magnification (can interfacial reaction be detected between SiO2 and YAG at the nanometer/atomic scale (TEM)?

**Reply 7:** We have conducted TEM measurements to study the interface reaction between YAG:Ce particles and glass matrix at nanometer scale. As shown in **Fig. R3a**, a clear boundary representing the interface can be observed, which is confirmed by the concurrence of the diffraction spots belonging to amorphous SiO_2_ and YAG:Ce crystal in the SAED pattern of the interface. In the TEM-EDS element spectra (**Fig. R3b**) the abrupt but opposite changes in the content of Y/Al and Si elements denote the interface reaction zone, and the thickness of the interface of YAG:Ce-PiSG is thus estimated to be about 150 nm. Considering the size of YAG:Ce particles is about 10~20 μm, only 2~4 vol% of the YAG particle is corroded by silica glass matrix, which is consistent with the IQE loss (~2.6%) of YAG:Ce after embedded into silica glass as well as our NMR data. In comparison, this thickness was about 250 and 130 nm (**Fig. R3c and d**) for YAG:Ce-PiGs using low-melting silicate glasses and Bi_2_O_3_ based borate glass, respectively; note that the sintering condition is **800 °C for 30 minutes** for the former, and only **325 °C for 30 minutes** for the latter, whereas **1250 °C for 3 hours** for our PiSGs. Furthermore, the as-synthesized YAG:Ce-PiSG shows no IQE loss even after they are heat-treated at 1200 °C in air for 10 hours. Our results clearly demonstrate that the interface reaction is well suppressed when silica glass is used as the matrix, as discussed in our manuscript. (**Fig. R3a and b** are also added in the revised manuscript)

**Fig. R3.** (**a**) TEM bright-field image (left) and SAED patterns of YAG:Ce-PiSG. (**b**) TEM-EDS line scanning of YAG:Ce-PiSG. (**c**) and (**d**) TEM bright-field images of YAG:Ce-PiGs based on low-melting silicate glass and Bi_2_O_3_ based borate glass, respectively; note that TEM images of YAG:Ce-PiGs were quoted from the literature (***Ref.:*** *J. Am. Ceram. Soc. 2017, 100, 1460-1471*).

**Question 8:** The fact that SiAlON is not as stable as the other examples might also be related to the fact that there is Si in the crystalline phase.

**Reply 8:** We agree with you that the existence of Si may also result in the limited stability of β-SiAlON:Eu^2+^ at high temperature. Besides, it should be noted that researchers have also tried to incorporate β-SiAlON:Eu^2+^ into low-melting glass (ZnO-B_2_O_3_-BaO-Al_2_O_3_), but still fails to achieve high QE (< 50%), where the sintering temperature is only 650 °C and the holding time is only 40 minutes, implying that β-SiAlON:Eu^2+^ is thermally unstable (***Ref.****: J. Mater. Chem. C, 2015, 3, 10761*).

**Question 9:** Minor comments:

The acronym (PiSG) should be developed at the first occurrence of the Phosphor in silica glass expression.

In the "Performance characterizations..." section: replace "As shown in Fig. 4a" by "As shown in Fig. 2a".

In conclusion the manuscript is a nice piece of work of which I recommend publication. Hopefully the manuscript can be further improved by answering the above comments/suggestions.

**Reply 9:** We are sorry for our mistakes, and we have corrected them all in our revised manuscript.

**Finally, we thank you for the constructive and valuable comments again.**

**Response to Reviewer #2:**

Dear Reviewer:

Thank you very much for your constructive and valuable comments on our manuscript. According to your suggestions, we have made relevant revision. The revised sentences were highlighted with red font color in the revised manuscript.

**General comment:** The manuscript suggests a new method to fabricate a phosphor in glass using nano-sized silica powders to embed phosphors. It seems to be reasonably written but the followings should be addressed or discussed before consideration of publication.

**Question 1:** They suggested that their method is cost-effective process compared to other previous methods. It could be true for phosphor in silica glass (PiSG) fabricated by SPS process. However, their method still require quite high sintering temperature of 1250 oC along with reducing atmosphere. When compared with commercial phosphor in glass (PiG) using silicate glass which is sintered at 750 oC without atmospheric control, the present method require much higher temperature and needs special facility to control atmosphere as well, demanding higher production cost. Even considering the cost of gas pressured sintering (GPS) for the densification of commercial PiG, it is hard to believe that the present method could be cost effective in mass production when considering the cost of nano-sized silica glass powders.

**Reply 1:** **First,** the sintering temperature used here is easily accessible to the tube furnace using SiC heater, which is 5~10 times cheaper than those furnaces for GPS and SPS. **Second,** the reducing atmosphere (5%H_2_+95%N_2_) is similar with (or even milder than) that of synthesizing Ce^3+^ or Eu^2+^ activated oxide phosphors, so that anyone can reproduce PiSG if they have a tube furnace used for phosphors before (gas regulator and barometer are also cheap); for example, the flow of the reducing atmosphere used in our preparations is about 0.1 L/min, which means a full gas cylinder (40 L, 12.5 Mpa, 70 dollars) of the 5%H_2_+95%N_2_ mixed gas can be used for 30~40 times. **Third,** it should be noted that amorphous silica nanoparticles (fumed silica) have been widely commercialized and used in many industrial fields including cosmetics, silicon rubbers, adhesives and heat insulators; therefore, its price is as low as traditional SiO_2_ powders; for example, it cost us only about 2500 RMB (360 dollars) to buy 15 kilograms of amorphous silica nanoparticles (a mean diameter of 40 nm, Aerosil OX50, Evonik, Germany). Obviously, the method presented here could be cost-effective in mass production.

**Question 2:** Although the authors suggested that the present PiSG showed high transparency, the highest transparency was ~ 80% in visible range for the sintered SG without phosphor. As they mentioned, it is due to the residual pores within the sintered silica glass. Since the pores cannot be avoided without vacuuming or external gas pressure during the sintering process, it is hard to maintain the same color coordinate in mass production, which is why commercial PiG goes under additional GPS process. They need to discuss how they can control the amount of pores and their distribution within the silica glass matrix for reproducible mass production. Moreover, in order to suggest the advantage of the present pressure-less method, they need to compare color conversion properties with a PiG which used silicate glass and GPS process.

**Reply 2:** The transparency of silica glass sintered here is significantly higher than that of other glasses sintered without high pressure. The amount of residual pores in our PiSG samples is very limited, which is comparable to other PiG samples obtained by GPS method or even SPS method (see **Fig. R4**). And unlike color centers the pores have no deleterious effect on the luminescent properties of PiSG. We also agree with you that completely avoiding residual pores is beneficial to the conversion properties of PiG composites; however, such improvement due to further reducing residual pores could be limited as the scattering effect in PiSG dominantly comes from the difference in refractive index between silica glass and YAG:Ce; therefore, we think it is not desirable to achieve theoretical transparency by complicated techniques which usually lead to high costs. Besides, as can be seen from **Fig. R4**, the pores appear mainly in the proximity of phosphor particles for all the three methods, implying the morphology of phosphors should be considered in controlling the pores. For reproducible mass production, we should ensure the homogeneous distribution of the temperature and atmosphere inside the furnace, and precisely control the uniformity of green bodies. To the best of our knowledge, PiGs based on silicate glasses and synthesized by GPS are seldom reported in the literatures. An important paper (***Ref.:*** *Opt. Lett. 2016, 41, 1590-1593*) reported that GPS can be used to achieve fully dense PiG plates embedded with Ca-α-SiAlON for automotive applications. It was also demonstrated that the IQE is reduced from **88.4%** to **74%** when Ca-α-SiAlON is embedded into silicate glasses (GPS at 710 °C for 30 min). Besides, Zhang *et al.* (***Ref.:*** *J. Mater. Chem. C, 2019, 7, 354*) have recently studied the color conversion properties of silicate glass based YAG:Ce-PiG with **IQE~70%** (**IQE 95%** for YAG:Ce-PiSG); they showed that the luminescence saturation of YAG:Ce-PiG is only about **0.5 W/cm^-2^** (**3.46 W/cm^-2^** for YAG:Ce-PiSG) without additional heat sink, and it can be improved to 1.21 W/cm^-2^ when sapphire plate was used as the heat sink. In addition, the researchers from Nippon Electric Glass (Japan) who pioneered the development and commercialization of PiGs ([*https://www.neg.co.jp/en/rd/topics/product-lumiphous/*](https://www.neg.co.jp/en/rd/topics/product-lumiphous/)) have also pointed out that when the silicate glass matrix containing alkali metal elements is irradiated with high-power excitation light, color centers can be easily formed, which will decrease the luminescence intensity of the PiGs (***Ref.:*** *US patent: US20190119148A1*). Obviously, our PiSGs have significant advantages over these PiGs. Finally, we also agree with you that among high-pressure assisted techniques GPS is superior to others like SPS and hot isostatic pressing for the synthesis of PiGs, and thus we have cited this method in the introduction part of our manuscript (***Ref.:*** *Opt. Lett. 2016, 41, 1590-1593*).

**Fig. R4.** **SEM images of PiSGs prepared by our pressureless method, GPS and SPS (left to right).** Note that SEM images of PiGs using GPS and SPS were quoted from the literatures (***Ref.:*** *J. Korean Ceram. Soc. 2019, 56, 71-76; Opt. Mater. Express 2017, 7, 4304-4315*). Red circle represents where the pores are located. Although low-melting glasses are used as the matrix and high pressure is applied in GPS and SPS methods, several pores still exist within these PiGs.

**Question 3:** As previous studies also reported PiSG with other methods such as sol-gel or SPS sintering, it seems reasonable to compare their data with the present method to clearly demonstrate their novelty.

**Reply 3: First,** in the sol-gel method (followed by sintering) tetramethoxysilane (metal alkoxide, expensive) is hydrolyzed and condensed in a solution, which suffers from easy formation of cracks during solvent evaporation (***Ref.:*** *Sci. Technol. Adv. Mater. 2011, 12, 034407*); therefore, supercritical drying (the wet gel is put into an autoclave and dried at a temperature of 80 °C and a high pressure of 20 MPa) should be used to avoid the capillary force during drying of the solvents (***Ref.:*** *Int. J. Appl. Glass Sci. 2017, 8, 247–252*); it takes at least several days to finally get the dried gels; as we know, the long-time heat-curing process of the sol-gel method will inevitably lead to strong phosphor sedimentation; moreover, through the sol-gel method they also failed to achieve high IQE of PiSGs (IQE< 50%), because of which, we think, they turned to searching for low-melting glasses as the matrix (***Ref.:*** *Ceram. Int. 2018, 44, 4783–4788*; *J. Eur. Ceram. Soc. 2018, 38, 735–741*). **Second,** previous studies demonstrated that low sintering temperature and short processing time should be fulfilled to inhibit the interface reaction between phosphors and glass matrix; it seems impossible to obtain highly efficient YAG:Ce-PiSG by using traditional sintering techniques, so that Zhou *et al.* adopted an advanced sintering technique, SPS to densify PiSG with a holding time of 3 minutes (otherwise, 30 minutes is needed for previous methods) (***Ref.:*** *Acta Mater. 2017, 130, 289–296*); however, such a high pressure assisted technique is not suitable for mass production as well as suffers from high cost; in addition, carbon contamination from graphite die is very difficult to be avoided during SPS processing (***Ref.:*** *J. Eur. Ceram. Soc. 2018, 38, 2588–2595; ACS Appl. Mater. Inter. 2018, 10, 5673−5681*); the presence of carbon increases the undesired light absorption, which causes larger IQE loss of PiSG (IQE loss: 6.5% for SPS, 2.7% for ours). **Most importantly,** in contrast to previous studies, here we demonstrate that interface reaction could be controlled even at high sintering temperature (and longer sintering time) if the constituents of the inorganic matrix is rationally designed, which will leads to better chemical and thermal stabilities of these composites without deteriorating the performance of the embedded particles; we believe our results will have strong implications for developing other composite materials.

**Question 4:** Although they successfully fabricated PiSG with various phosphors, due to high sintering temperature of 1250 oC, only limited phosphors which have very high thermal stability can be applied. For example, as they indicated, beta-SiAlON showed degradation of IQE. This suggests that their method cannot incorporate other phosphors such as CaAlSiN-based red phosphors and thus their application could be limited.

**Reply 4:** We admit that CaAlSiN_3_:Eu^2+^ and Sr_2_Si_5_N_8_:Eu^2+^ cannot be incorporated into silica glass by our method or any other strategies because of their poor stability. We are also aware that these red phosphors show obvious IQE loss even when embedded into low-melting glasses (***Ref.:*** *J. Alloy. Comp. 2017, 702, 193–198; J. Am. Ceram. Soc. 2019, 102, 1259–1268*), and only by using ultra-low melting glasses like Sn-P-F-O glass (***Ref.:*** *J. Eur. Ceram. Soc. 2018, 38, 1990–1997*) can they retain high IQE; however, these low-melting glasses possess low transparency and limited chemical and thermal stabilities, and may contains some expensive and/or toxic elements. The most commercialized phosphor-converted white LEDs (pc-WLEDs) are fabricated by using YAG:Ce yellow phosphor as the converter. The resulting pc-WLEDs usually have high CCT (> 5500K) and low *R*_a_ (~70) owing to the deficiency in red emission of YAG:Ce. This problem can be mitigated to some extent by adding orange GdAG:Ce phosphor to achieve CCT < 5000 K and *R*_a_ ~80 (***Ref.:*** *ACS Appl. Mater. Inter. 2019, 11, 2130−2139*). Note GdAG:Ce embedded in silica glass by our method is also highly efficient. Of course, for warm pc-WLEDs with ultra-high *R*_a_ (> 90) and low CCT (< 4000 K), red phosphors are indispensable to further enrich the red emission (＞ 610 nm). Therefore, we believe this limitation cannot be well circumvented until novel oxide or oxynitride red phosphors with high thermal stability are developed, which requires tremendous research effort.

**Question 5:** The authors suggested that the reaction between SiO2 and YAG has been suppressed based on SEM/EDS and NMR results. However, other previous results which used silicate glasses also showed no apparent reaction layers between the glass matrix and phosphors based on SEM/EDS results. Moreover, since reaction is mostly restricted in the interface region and NMR signal gives averaged effect within the composite, it is hard to identify the possible reaction between the glass and phosphor by monitoring Al-NMR. It seems the phosphor-glass reaction has been suppressed since it was sintered under reducing atmosphere, but in order to clearly claim it, it seems to show TEM result as reported previously (Shyu et. al, J. Am. Ceram. Soc., 100, 1460-1471 (2017)).

**Reply 5: First,** as we have mentioned in the manuscript, previous studies showed that the interface reaction between phosphors and glass matrix could be limited only if low-melting glasses are used and the sintering time is shorter than about 40 minutes. In contrast, our YAG:Ce-PiSG was sintered at **1250 °C for 3 hours**, and the interface reaction is still very limited. Hence, our results are intrinsically different from previous studies. **Second,** although the reaction is restricted within the interface region, the NMR signals could give a full view of the interface reaction within the PiSG composite if the interface reaction is strong, which is complementary to the SEM/TEM analysis. **Third,** to study the interface reaction between YAG:Ce particles and glass matrix at nanometer scale, we have conducted TEM measurements. As shown in **Fig. R3a**, a clear boundary representing the interface can be observed, which is confirmed by the concurrence of the diffraction spots belonging to amorphous SiO_2_ and YAG:Ce crystal in the SAED pattern of the interface. In the TEM-EDS element spectra (**Fig. R3b**) the abrupt but opposite changes in the content of Y/Al and Si elements denote the interface reaction zone, and the thickness of the interface of YAG:Ce-PiSG is thus estimated to be about 150 nm. Considering the size of YAG:Ce particles is about 10~20 μm, only 2~4 vol% of the YAG particle is corroded by silica glass matrix, which is consistent with the IQE loss (~2.6%) of YAG:Ce after embedded into silica glass. In comparison, this thickness was about 250 and 130 nm (**Fig. R3c and d**) for YAG:Ce-PiGs using low-melting silicate glasses and Bi_2_O_3_ based borate glass, respectively; note that the sintering condition is **800 °C for 30 minutes** for the former, and only **325 °C for 30 minutes** for the latter, whereas **1250 °C for 3 hours** for our PiSGs. Furthermore, the as-synthesized YAG:Ce-PiSG shows no IQE loss even after they are heat treated at 1200 °C in air for 10 hours. These results clearly demonstrate that the interface reaction is well suppressed when silica glass is used as the matrix. (**Fig. R3a and b** are also added in the revised manuscript)

**Fig. R3.** (**a**) TEM bright-field image (left) and SAED patterns of YAG:Ce-PiSG. (**b**) TEM-EDS line scanning of YAG:Ce-PiSG. (**c**) and (**d**) TEM bright-field images of YAG:Ce-PiGs based on low-melting silicate glass and Bi_2_O_3_ based borate glass, respectively; note that TEM images of YAG:Ce-PiGs were quoted from the literature (***Ref.:*** *J. Am. Ceram. Soc. 2017, 100, 1460-1471*).

**Finally, we thank you for the constructive and valuable comments again.**

**Reviewer comments, second version:**

REVIEWERS' COMMENTS:

Reviewer #1 (Remarks to the Author):

The authors have provided detailed answers to the different points I raised previously. Overall I am satisfied with their answers and advise publication of their manuscript.

Regarding the interface reaction between SiO2 and YAG, it is not "suppressed" as explained in the rebuttal letter (reply 7) but "limited" as stated in their manuscript. Full suppression would be of great interest for the development of similar materials with YAG nanocrystals (in fibers for example). But it's not the case here as demonstrated by the authors. Hopefully it will be one day...

Given the answers provided by the authors and the modification they brought to the manuscript, I recommend publication of the manuscript as it is now.


Reviewer #2 (Remarks to the Author):

The manuscript has been improved and the queries seem to be responded properly. It seems suitable for publication after some minor considerations.

1. The authors should know that the commercial PiG products should have no pores inside to avoid 'binning (change of color properties depending on lot#)'. Thus, pore removing is crucial for its commercialization and needs to be briefly addressed for their possible strategies.

2. As stated in their response letter, PiSG cannot be applied to the phosphors with relatively low thermal stability. Thus, it seems better to address its limitation within the manuscript.

**Author rebuttal, second version:**

**Response to Reviewer #1:**

Dear Reviewer:

Thank you very much for the positive recommendation.

**Comment:** The authors have provided detailed answers to the different points I raised previously. Overall I am satisfied with their answers and advise publication of their manuscript.

Regarding the interface reaction between SiO2 and YAG, it is not "suppressed" as explained in the rebuttal letter (reply 7) but "limited" as stated in their manuscript. Full suppression would be of great interest for the development of similar materials with YAG nanocrystals (in fibers for example). But it's not the case here as demonstrated by the authors. Hopefully it will be one day...

Given the answers provided by the authors and the modification they brought to the manuscript, I recommend publication of the manuscript as it is now.

**Reply:** It is possible to realize full suppression if using smaller amorphous silica nanoparticles (< 15 nm) or tetramethoxysilane and adding amorphous GeO_2_ or TiO_2_ nanoparticles to reduce the sintering temperature to lower than 1100 °C, as we mentioned in our manuscript. However, it is another challenging work to develop suitable process for obtaining highly transparent glasses.

**Response to Reviewer #2:**

Dear Reviewer:

Thank you very much for your constructive and valuable comments on our manuscript. According to your suggestions, we have made relevant revision. The revised sentences were highlighted with red font color in the revised manuscript.

**General comment:** The manuscript has been improved and the queries seem to be responded properly. It seems suitable for publication after some minor considerations.

**Question 1:** The authors should know that the commercial PiG products should have no pores inside to avoid 'binning (change of color properties depending on lot#)'. Thus, pore removing is crucial for its commercialization and needs to be briefly addressed for their possible strategies.

**Reply 1:** For its commercialization there is much optimization work to be done. As regards removing residual pores, we should further optimize the processing of composite slurry containing silica nanoparticles and YAG:Ce powders, so that there is no bubbles in the resulting slurry. Note that some small bubbles induced by the stirring (indispensable step for obtaining homogeneous slurry) may still exist despite the defoaming by a vacuum ball mill. And the sintering atmosphere, the heating rate as well as the holding time should be also further optimized. Besides, the morphology (spherical geometry is preferred) and surface of phosphor particles may influence the formation of pores, which deserves investigation. Finally, we believe it is highly possible to further improve the transparency of PiG without using high-pressure and high-vacuum sintering techniques, which will benefits the cost cutting of PiG products.

**Question 2:** As stated in their response letter, PiSG cannot be applied to the phosphors with relatively low thermal stability. Thus, it seems better to address its limitation within the manuscript.

**Reply 2:** We have addressed this limitation in the last paragraph of the discussion part of our revised manuscript.

**Finally, we are grateful for your constructive and valuable comments.**

**Reviewer comments, third version:**

**Author rebuttal, third version:**
